# Decoding the Behavior of a Queenless Colony Using Sound Signals

**DOI:** 10.3390/biology12111392

**Published:** 2023-10-31

**Authors:** Dimitrios Kanelis, Vasilios Liolios, Fotini Papadopoulou, Maria-Anna Rodopoulou, Dimitrios Kampelopoulos, Kostas Siozios, Chrysoula Tananaki

**Affiliations:** 1 Laboratory of Apiculture-Sericulture, Aristotle University of Thessaloniki, Aristotle Farm, 57001 Thermi, Greece; dkanelis@agro.auth.gr (D.K.); vliolios@agro.auth.gr (V.L.); foteinpi@agro.auth.gr (F.P.); rodopoum@agro.auth.gr (M.-A.R.); 2 Section of Electronics and Electronic Computers, Department of Physics, Faculty of Sciences, Aristotle University of Thessaloniki, University Campus, 54124 Thessaloniki, Greece; dkampelo@physics.auth.gr (D.K.); ksiop@auth.gr (K.S.)

**Keywords:** queenright state, queenless state, mel bands, honeybee colony

## Abstract

**Simple Summary:**

In the last decade, a large decline of honey bee colonies has been observed, caused by multiple factors that may affect them and contribute to their total extinction. Therefore, it is of the utmost importance to find successful ways of extensively monitoring beehives’ status in order to detect potential threats. In this context, one of the states of a bee colony that should be monitored is the queenless state, so in the present research, we tried to investigate the behavior of honeybees during their queen’s loss through recording and analyzing sound data from beehives over a certain period.

**Abstract:**

Honeybees are reported to be the most vital pollinators of agricultural and horticultural crops. However, their widespread decline has raised great attention to the need to monitor their activity in order to identify the causes and implement countermeasures. The recording and analysis of signals used by honeybees for their communication in their hive can be a very helpful tool to the beekeepers for the remote control of the hives. Thus, in the present study, we used a set of sound recording data taken inside the hives to automatically detect the sounds of the bees over a certain period, to distinguish between queenright and queenless states, and to find the gradual changes in the queenless state. Unlike what was commonly believed, noticeable changes in the sound signals of all experimental colonies were observed just one hour after the queens’ removal from the hive, while the sound signals were intensified over a period of 5 h, after which the transmitted signal stabilized to the equivalent of a queenless state. The colonies seemed to return to their normal sounds 9–10 days after the reintroduction of the queens in the hives. Our study concluded that timely intervention of the queen’s absence combined with the immediate intervention of the beekeeper may be a determining factor in mitigating the adverse effects that occur from the queen’s loss.

## 1. Introduction

The honeybee (*Apis mellifera* L.) is one of the most common managed pollinators, providing its pollination services for a multitude of crops, contributing to the well-being of ecosystems around the world. It is estimated that it is responsible for the pollination of over 90% of global commercial pollination services, and approximately 35% of the world’s food crops [1]. In the last few years, there has been a drastic decrease in bee colonies around the world, caused by multiple factors that act independently or in combination and that affect the health of honeybees [2,3]. Monitoring the status of beehives is therefore crucial for the survival of honey bee colonies, and understanding how bees behave during a queenless state can provide valuable insights into potential threats to the colony. The recording and analysis of sound data emitted by honeybees living in a hive is a novel approach that could offer new insights into bee behavior, while their accurate interpretation can determine and predict specific conditions. The most important states that are mainly examined in the literature include the presence and absence of the queen (queenless colonies), the abandonment of a hive by the old queen (swarming) and the occurrence of various diseases.

It has been reported that honeybees produce a variety of rhythmic thoracic oscillations, which are transmitted to potential receivers as substrate vibrations or as air borne sounds, as a form of communication within the hive [4]. Gross body movements, wing movements, high-frequency muscle contractions without wing movements, and pressing the thorax against the substrates or another bee are just some of these vibroacoustic signals present within a hive [5]. Moreover, vibroacoustic signals generated by bees modulate behaviors that affect swarming, as well as the queen’s behavior during swarming. In this regard, research has shown correlations between the frequencies of the vibroacoustic signals and their amplitudes with events such as swarming [6]. Indeed, the “sound” of a colony is a mixture of individual signals emitted by each honeybee, perceived by the beekeepers as a dense, continuous and low frequency buzz [6]. According to Woods [7], the worker bee produces hums at a frequency of 255 ± 35 Hz [8], while, as stated in the existing literature, most sounds are produced in a hive range from 100 to 1000 Hz with their respective harmonics [3,9,10,11], with the majority of sound frequencies being around 300, 410 and 500 Hz [12]. Various sounds originating from the inner of the hive can be the source of important information, while they may correspond to a range of sounds of different acoustic frequencies emitted by honeybees for a variety of reasons [1]. However, the acoustic structure in terms of signal pattern may also affect, along with the range of frequencies, the final meaning of the noise; thus, the quantification of both characteristics is the key to the development of a system that can recognize possible threats to the bee colonies [1].

Earlier measurements trying to interpret honeybees’ signals, include endoscopic observations and calorimetric traces, the long-term study of vibrations recorded by accelerometers and measurements of electric fields [13,14,15]. Regarding sound data recording, in previous studies, during the pre-processing of the signal, a band-pass filter was applied in the first stage, with the cut-off frequency of the high frequencies being 2 kHz, while for the cut-off frequency of the low frequencies, there were options from 20 to 100 Hz [8,16]. Apart from the various filtering techniques, the wavelet decomposition and spectral subtraction techniques are also applied [17]. In turn, the use of MFCCs (Mel Frequency Cepstral Coefficients) is proposed for the extraction of the characteristics of the signal waves for their representation (inspired by speech recognition) [18]. However, for the extraction, other techniques are also used, such as HHT (Hilbert Huang Transform) combined with Wavelet analysis [3], LPC (Linear Predictive Coding) [16], STFT (Short-time Fourier Transform), Mel spectrogram, spectral contrast features and chroma-features [19].

One of the states mentioned above that should be detected and controlled is that of the queenless colony. The queen bee is crucial to the survival and productivity of the honeybee colony, as she is responsible for maintaining the cohesion of the colony through the use of pheromones. In the event of queen loss, the colony’s productivity can decrease and, if a new queen is not introduced, the colony may ultimately perish. Regularly checking the health of the queen and replacing her if necessary is an important task for beekeepers in order to maintain the health and productivity of the colony.

Several authors give different time intervals that honeybee workers start to construct new queen cells, such as the first 3–4 h, 10 h, 12–48 h, or even 9 days after queen’s loss [20,21,22,23,24]. This variability in the time may not indicate precisely when a colony begins its transition to the queenless state [25]. Thus, the absence of the queen in a beehive requires beekeeper’s intervention to save its colony from a possible loss, based on their experience; beekeepers usually perceive a queen’s loss from a colony due to produced specific sounds and the colony’s disorganized activity [26]. However, should they realize the orphan honeybee colony in the last stages of the state, it would result in delays for its control. Also, daily hive inspections and checking whenever queen bee is present disrupts the normal life cycle of the bee colony and can be a source of stress for honeybees, while at the same time it can be the cause of accidental queen death.

To avoid such situations, it is necessary to use a non-invasive method that is able to detect the lack of the queen and help beekeepers intervene in a timely manner. Researchers have been analyzing different parameters by using sensors, such as temperature, humidity, vibrations, weight and sound, to determine the colony state [9,27,28,29,30]. Also, sound analysis systems have been primarily studied, leading to distinct patterns between queenright and queenless colonies, without, however, indicating the time of the change of the state, or the progress of the queenless state [13,31,32].

Considering all the above, the aim of the present study was, through a set of sound recording data taken inside the hives, to automatically detect the sounds of the bees and distinguish between the queenright and the queenless states over a certain period, and find the gradual changes in the queenless state. In addition, the deciphering of behavioral patterns of honeybee behavior through audio recordings will practically contribute to the optimization of production as well as the reduction in relevant cost.

## 2. Materials and Methods

### 2.1. Honeybee Colonies

The study was conducted in the experimental apiary of Apiculture-Sericulture, AUTH, located in Thessaloniki, an area mostly characterized as a suburban environment. To artificially induce bee states, such as the absence of the queen (queenless colonies), we used five bee colonies of *Apis mellifera macedonica* with equal populations. All the five bee colonies, had queens from the current beekeeping year to avoid the effect of queens’ age on the sound signals of the colonies [16]. Beekeeping practices were applied to trigger these states and observe the behavior of the bees before and after the reintroduction of the queen. To ensure that the sound recording of bees’ behavior is not affected by differences in population status, we inspected the beehives and recorded their capacity on recording sheets. Specifically, we visually estimated comb surfaces covered by brood and adult workers and considered a population frame when ¾ of the frame was covered by bees. Then, we carried out transfers of sealed brood frames to and from the experimental colonies to achieve the desired strength. All transfers were carried out at least 20 days before the start of the experiment so that the colonies came to rest. Additionally, we determined the reserves in food (honey and pollen) and brood in the same way to ensure equivalence among the bee colonies.

### 2.2. Beekeeping Practices to Trigger the State

The whole experiment started in the spring and ended in the fall. During this time, the acquisition system was successively transferred between the five experimental bee colonies. In order to achieve the required recordings of a strong bee colony, the microphones were placed above the queenright colony to capture the physical behavior of honeybees over an extended period. The colonies were inspected daily before removing the queens to ensure their natural presence. The sound background of each hive was recorded for 5 days prior to queen removal. The recordings continued for 10 days, after which the queens were reintroduced in the hive using queen cages. During the period of queens’ nonappearance, the colonies were inspected daily to confirm the presence of unsealed queen cells, indicating the loss of the queen. After the queens’ return, the recordings continued in this state for the next 15 days while inspecting the colonies for normal queen laying behavior. Continuous recording was conducted, with audio files downloaded every 30 min. The distinction between the queenright and queenless states was achieved by analyzing the sound data from the beehive recordings over an extended period of time.

### 2.3. Recording System/Sound Analysis

The acquisition system for each hive consisted of a set of two microphones (Behringer ECM8000, Willich, Germany), recording at a sampling rate of 44.1 kHz and a 16-bit resolution, and connected via a wire to a computer’s external sound card (Focusrite Scarlet 8i6, High Wycombe, UK). The system was also equipped with a set of temperature and humidity sensors (BME280) that were installed both inside and outside the hive to monitor the environmental and the internal conditions of the hive. These sensors were connected to a Raspberry Pi used to handle the acquisition and upload the data to the online database. The general process that was followed involves Mel’s Frequency Transformation and the extraction of a set of features from the raw audio data. In this process, the frequency content of the sound is explored, and the spectrum is divided into a number of frequency bands (mel bands). The power exhibited in these bands was calculated for different days of measurements. More specifically, a set of features was extracted—Mel’s Frequency Cepstral Coefficients (MFCCs). The values of these features are the key to make the distinction between the different states of the experiment. The concept is that the bees generate different frequency components depending on their state, altering the power distribution between the bands.

All the steps of the data analysis process were performed in Python, utilizing the Scipy and the Librosa libraries for the pre-processing and feature extraction stages. First, the audio data are resampled from the initial sampling frequency down to 4096 Hz. The data are then pre-emphasized with a 0.97 ratio and are filtered through a band-pass Finite Impulse Response (FIR) filter at a frequency range of 80 Hz–2 kHz to eliminate the noise components generated by the supply circuit (60 Hz) and any residual higher frequencies. For the MFCC features, a set of nine mel bands (#0–#8) was selected (Table 1).

The features were calculated for each second of audio recording by averaging through short duration windows. As a result, for each second, there are nine numerical values, each corresponding to a specific mel band on the spectrum. The probability distributions of these values for different days or hours of the experiment were used to visually indicate the distinction between the different states.

## 3. Results and Discussion

**Figure 1:**
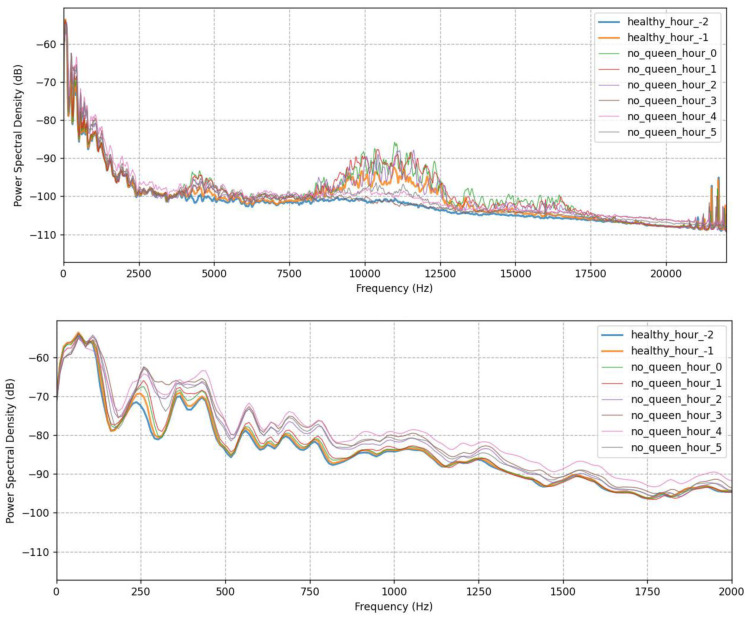

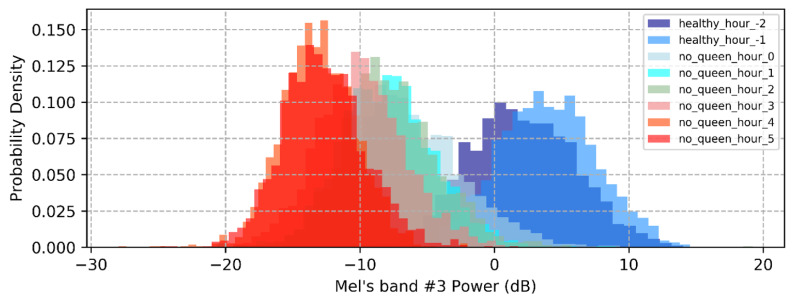
Power Spectral Densities for the first hours after the removal of the queen and the distributions for mel band #3.

The behavior of bee colonies and the changes that occur after the removal of their queen was studied through sound signals recording over a period of 6 months. The perception that prevails among beekeepers until now is that honeybees notice the loss of their queen within a timeframe of 6 h, while the international literature does not define a clear interval for when the queenless state start to develop. In the present study, however, we observed a noticeable change in the sound signals of all experimental colonies within just one hour of the queens’ removal from the hive. Indeed, further analysis of the spectral content and processing of the Mel bands revealed the intensification of the sound signals over a period of 5 h, after which the transmitted signal stabilized to the equivalent of a queenless state. The power spectral density in dB is given in Figure 1 for data acquired two hours before and five hours after the queen’s removal. Notice that there are spectral components throughout the spectrum, but when focusing on the range below 2 kHz, the two states are clearly distinguishable. In Figure 1, the distributions for mel band #3 are also given as an indication of how a specific mel band is distributed as the phenomenon progresses. Apart from that band, mel bands #1 and #5 exhibited the same behavior. It seems that large fluctuations in sound signals occur at frequencies lower than 1080 Hz, which is consistent with the reported range of beehive sounds (100–1000 Hz) with the respective harmonics.

**Figure 2:**
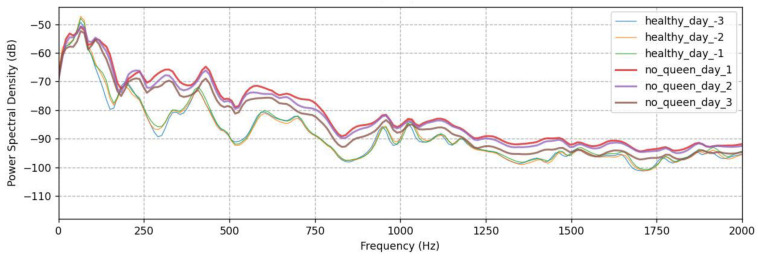

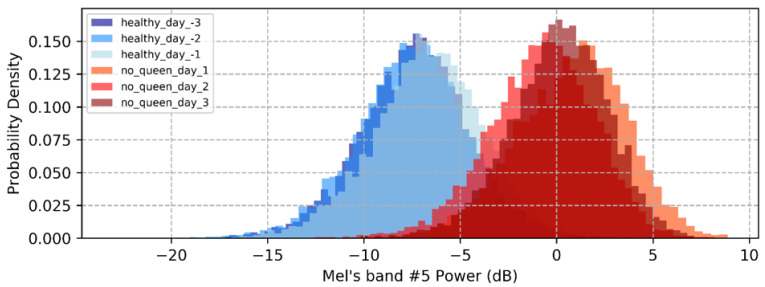
Power Spectral Densities for three days before and after the removal of the queen and the distributions for mel band #5.

Thus, it appears that honeybees are able to recognize the loss of their queens from the first instance of their absence, a finding that, according to our knowledge, has not been previously reported in the literature. Overall, the absence of queens led to a gradual change in the produced frequencies, which became lower and more intense, essentially resulting in the honeybees becoming noisier, and then the signal remained constant regardless of the recording day. Indeed, during inspections we conducted in the experimental colonies at these time intervals, a noticeable buzz (an outcry) began to develop inside the hive and continued as long as the queen remained isolated, while an intense mobility in the hive was observed, as honeybee workers began constructing new queen cells. The same behavior was observed in all experimental honeybee colonies, with differences being observed in the same mel bands #1, #3, and #5, and within the same time frame, regardless of the season. The sound signals remain stable for each particular state, as shown in Figure 2, illustrating the spectrum and mel band’s #5 distribution of the colonies 3 days before and after the removal of the queens, respectively. There are distinct differences in the spectrum between the two states, while data from the same state are almost identical in terms of spectral signature and mel band distributions.

**Figure 3:**
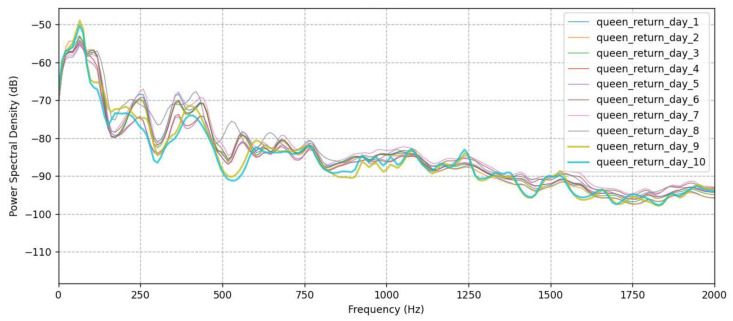

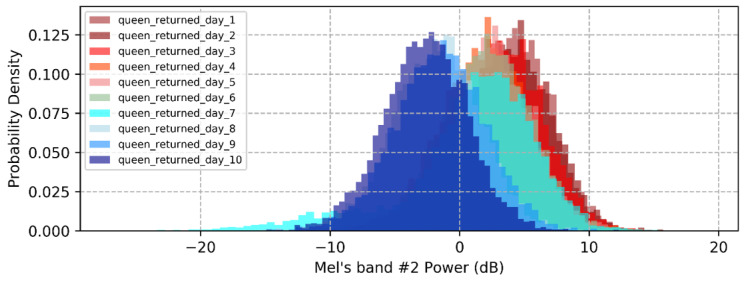
Power Spectral Densities for the first 10 days after the queen was returned into the hive and the distributions for mel band #2.

In all experimental honeybee colonies, the queen was reintroduced to their hives 10 days after her removal, and her successful release inside the hives was confirmed. All queens continued their oviposition 2–3 days after their release from the queen cages. Indeed, during the inspections we conducted 3 days after the reintroduction of the queens, we observed that the mobility of honeybee workers had calmed down, while we no longer heard the outcry of honeybee workers. However, according to the sound signals from the microphones and the processed data, the colonies appeared to return to their normal sounds 9–10 days after the reintroduction of the queens in the hives, suggesting that the beekeepers should wait approximately 9–10 days before proceeding to beekeeping treatments in a queenright colony in order to avoid adverse effects on the queen’s health. This is indicated in Figure 3, where the Power Spectral Densities are depicted for 10 consecutive days after the queen is re-turned. While this is not clearly visible from the spectrum, it is indicated clearly by the distributions of mel band #2 that change noticeably for days 9 and 10.

Throughout the whole experiment the microclimate inside the beehives (temperature, humidity, etc.) seemed to remain stable.

The Mel scale is a non-linear transformation of frequency that emphasizes lower frequencies and de-emphasizes higher frequencies, making it useful for tasks where the emphasis is often on the lower frequencies, as in the case of honeybees. The Mel frequencies for the identification of bees’ sounds have been used by other authors, as well, showing good results regarding the identity of sound and the state of the colony, succeeding in distinguishing the behavioral patterns [11,33,34]. Indeed, Cejrowski and Szymański [34] noted that honeybees change their behavior according to varying external conditions and time, which can be detected using the MFCC features with SVM classifier, while Terenzi et al. [35] described the Mel-spectrogram and spectrogram features as the most promising for the queenless detection task. However, to our knowledge, this is the first time it has been observed that honeybees recognize the loss of their queens from just the first instance of their absence. Also, the colonies seem to return to their normal sounds 9–10 days after the reintroduction of the queens in the hives, contrary to the findings of Cejrowski et al. [16], that the colony did not return to the same state even 15 days after new queen bee insertion.

The queen bee is the central figure in a hive, serving as the mother of all other members and holding the colony together. Without her, the hive would fail and the bees would scatter. The queen is selected before birth and given a distinct egg cell and special diet during her larval stage, much like human royalty. After mating several times, the queen spends her life laying eggs and never leaves the hive again. The queen worker polymorphism and determination of female castes are based on differential larval feeding and remarkable phenotypic plasticity. Although honey bee queens develop from genetically identical fertilized eggs as workers, they grow faster, are larger, live longer and exhibit different behavior. A queen bee typically lives for two to five years and may leave with half the population to start a new hive if the colony becomes too large. If the queen is accidentally killed by a beekeeper or disease, the hive will be thrown into chaos until another queen is raised by the workers. The existence of honeybees and queens in a colony is crucial for its consistence; thus, a queenless state may result in the death of an entire colony within merely a few months unless a new queen is introduced. That is why both unhealthy queen and queenless states are extremely unfavorable and should be recognized as soon as possible to adopt proper beekeeping management strategies [36]. Our study has concluded that the timely warning of the queen’s absence combined with the immediate intervention of the beekeeper, may be a determining factor in avoiding the adverse effects that occur from the queen’s loss. The analysis of sound signals can be a very useful tool in understanding phenomena related to the honeybees’ behavior and even lead to a shift of established perceptions based on previous studies which did not use specialized recording systems.

The study of beehives through acoustic measurements has provided valuable insights into the behavior of bees. Fahrenholz et al. [13] have found that locomotor activities can be accurately captured through the acoustic frequency spectrum and were in good agreement with visual observations and calorimetric traces. Another study [37] focused on the characteristics and temporal distribution of vibrational signal, known as the “whooping signal,” which were recorded using accelerometers placed inside honey bee colonies (*Apis mellifera*) in France and the United Kingdom [14]. Additionally, sensors in the antennae joints were identified as mechanoreceptors, and laser vibrometry revealed that electric fields, along with sound, influenced the movement of the electrically charged flagellum [15]. Sound analysis was used to monitor the learning process of worker bees (*Apis mellifera*), where they responded to an electric shock with a hissing response characterized by loud sounds and vibrations [38]. Further research explored the relationship between hissing onset/offset times and sunrise/sunset, indicating that honeybees hiss predominantly during daylight hours, with the highest frequency at dawn [39]. Therefore, different conditions or states in a hive result in bees producing different sound signals, which can provide useful information for beekeepers if interpreted appropriately.

## 4. Conclusions

All things considered, it seems that a non-invasive method of hive monitoring through the recording and analysis of signals produced from honeybees is considered desirable. It can provide valuable insights into the behavior of honeybees without causing stress to the colonies. Also, the deciphering of behavioral patterns of honeybees through audio recordings will practically contribute to the optimization of production as well as the reduction in relevant cost. According to the results of the present study, in the case of the queen’s absence, from the first time, there is observed a large amplitude of intensity at low frequencies, and the honeybees become noisier, while 9–10 days after the reintroduction of the queen in the hive, the colonies seem to return to their normal sounds. Further research on analyzing signals from honeybees can provide valuable information on their behavioral patterns and contribute to their successful management and survival.

## Figures and Tables

**Table 1:** Mel bands range (Hz) from the divided spectrum.

Mel Band	Range (Hz)	Mel Band	Range (Hz)
#0	0–80	#5	876–1080
#1	80–279	#6	1080–1326
#2	279–478	#7	1326–1629
#3	478–677	#8	1629–2000
#4	677–876		

## Data Availability

Data supporting reported results are stored at the Laboratory of Apiculture-Sericulture, AUTH.
